# Clinical spectrum of rhabdomyolysis presented to pediatric emergency department

**DOI:** 10.1186/1471-2431-13-134

**Published:** 2013-09-03

**Authors:** Chun-Yu Chen, Yan-Ren Lin, Lu-Lu Zhao, Wen-Chieh Yang, Yu-Jun Chang, Kang-Hsi Wu, Han-Ping Wu

**Affiliations:** 1 Division of Emergency Medicine, Department of Pediatrics, Changhua Christian Hospital, Changhua, Taiwan; 2 School of Medicine, Chung Shan Medical University, Taichung, Taiwan; 3 Department of Emergency Medicine, Changhua Christian Hospital, Changhua, Taiwan; 4 Department of Biological Science and Technology and Institute of Biochemical Engineering, National Chiao Tung University, Hsinchu, Taiwan; 5 Department of Pediatrics, Taipei Tzuchi Hospital, the Buddhist Medical Foundation, Taipei, Taiwan; 6 Laboratory of Epidemiology and Biostastics, Changhua Christian Hospital, Changhua, Taiwan; 7 Department of Pediatrics, China Medical University Hospital, Taichung, Taiwan; 8 School of Chinese Medicine, China Medical University, Taichung, Taiwan; 9 Department of Pediatrics, Taichung Tzuchi Hospital, the Buddhist Medical Foundation, Taichung, Taiwan; 10 Department of Medicine, School of Medicine, Tzu Chi University, Hualien, Taiwan

**Keywords:** Rhabdomyolysis, Emergency department, Children

## Abstract

**Background:**

Rhabdomyolysis is a potentially life-threatening syndrome that can develop from a variety of causes. The aim of the work is to analyze the clinical spectrum and to evaluate the prevalence of various etiologies in children, who present to the emergency department (ED) with rhabdomyolysis.

**Methods:**

During a 6-year study period, we retrospectively analyzed the medical charts of patients, aged 18 years or younger, with a definite diagnosis of rhabdomyolysis and serum creatinine phosphokinase (CK) levels greater than 1000IU/L. We analyzed the clinical spectrum and evaluated the potential risk factors of acute renal failure (ARF).

**Results:**

Thirty-seven patients (mean age = 10.2 ± 5.5 years), including 26 males and 11 females, were enrolled in the study. Two of the most common presented symptoms in these 37 patients were muscle pain and muscle weakness (83.8% and 73%, respectively). Dark urine was reported in only 5.4% of the patients. The leading cause of rhabdomyolysis in the 0- to 9-year age group was presumed infection, and the leading cause in the 10- to 18-year age group was trauma and exercise. The incidence of ARF associated with rhabdomyolysis was 8.1 % and no child needed for renal replacement therapy (RRT). We did not identify any reliable predictors of ARF or need for RRT.

**Conclusions:**

The classic triad of symptoms of rhabdomyolysis includes myalgia, weakness and dark urine are not always presented in children. The cause of rhabdomyolysis in younger age is different from that of teenager group. However, the prognosis of rhabdomyolysis was good with appropriate management.

## Background

Rhabdomyolysis is characterized by skeletal muscle breakdown with leakage of muscle contents, including electrolytes, myoglobin, and other sarcoplasmic proteins (e.g. creatine kinase, lactate dehydrogenase, alanine aminotransferase, and aspartate aminotransferase) into the circulation [1,2]. Its major causes in children, include infections, trauma, exertion, drugs, toxins, metabolic disorders and electrolyte disorders [2-5]. Typically, rhabdomyolysis patients present with muscle pain, weakness, and reddish-brown urine. Nevertheless, the severity of rhabdomyolysis varies from an asymptomatic increase in creatine phosphokinase (CK) to heavy complications, such as acute renal failure (ARF), cardiac arrhythmias, compartmental syndrome, hypovolemic shock and disseminated intravascular coagulopathy [4-10].

As we know, ARF is the most recognized complication of rhabdomyolysis. In a larger study of 191 children with a CK level greater than 1000 IU/L who were sent to the emergency department (ED), the prevalence of ARF was 5% [11]. However, after the search of the related literature, it would appear that research discussing rhabdomyolysis in children that also presented to the pediatric ED is still rare. Therefore, in this study, we analyzed the clinical spectrum and prevalence of various etiologies in children with rhabdomyolysis that presented to the pediatric ED.

## Methods

### Patient population

This study was a single-center medical chart review of patients aged 18 years or younger who presented to the ED with a diagnosis of rhabdomyolysis based on their medical histories and elevated serum creatine kinase (CK) levels (>1000 IU/L) within 72 hours after admission to the ED. We identified potentially eligible patient visits by searching the Changhua Christian Hospital health records database. We selected charts with any of the following search terms in the primary or secondary discharge diagnosis fields: rhabdomyolysis (*ICD-9* 728.88), infective myositis (*ICD-9* 728.0), myalgia and myositis (*ICD-9* 729.1), and myoglobinuria (*ICD-9* 791.3). We reviewed 445 medical charts of all eligible patients during a 6-year period from January 2006 to December 2011. Four hundred and eight patient charts were excluded (for having initial serum CK levels < 1000 IU/L or showing a documented history of muscular dystrophy or other metabolic muscle disorder, a history of myocardial infarction, a history of chronic kidney disease, rhabdomyolysis that developed after admission to the hospital due to a coexisting condition or iatrogenic complication, or ages greater than 18 years). In total, 37 (8.3%) of the 445 patients were included into this series. The study was approved by the Institutional Review Board of Changhua Christian Hospital.

## Methods

Information about related clinical factors that was potentially causative for rhabdomyolysis were reviewed from the medical records of patients who met the inclusion criteria. The following information was obtained from the medical records of each patient: age, gender, family history, associated symptoms and signs (fever, localized muscle pain, dark urine, muscle weakness or altered mental status, muscle swelling and history of upper respiratory tract infection), laboratory tests [initial and peak serum white blood cell (WBC), hemoglobin (Hb), CK level, electrolytes, blood urea nitrogen (BUN), creatinine (Cr), lactic dehydrogenase, alanine and aspartate aminotransferase levels, myoglobin levels], and urine toxicology screens for amphetamine, opiates, barbiturates, and benzodiazepines. Other clinical data included fluid administered within the first 24 hours, bicarbonate therapy, development of renal failure, need for renal replacement therapy (RRT), length of hospital stay, death, and final causes of the rhabdomyolysis and ARF. We also evaluated follow-up data, including CK, Cr, myoglobulin, and any documentation of renal sequelae.

Rhabdomyolysis was defined as serum CK greater than 1000 IU/L in the absence of any previous cardiac etiology or genetic muscular dystrophy. Acute renal failure was defined as a serum creatinine level of more than the 97.5^th^ percentile for the patient’s age and gender [12].

Renal replacement therapy (RRT) was defined as the use of either peritoneal dialysis, hemodialysis (HD) or continuous renal replacement therapy (CRRT). We analyzed the clinical spectrum and prevalence of various etiologies in children with rhabdomyolysis presented to the ED and also evaluates the potential risk factors of ARF.

### Statistical analysis

Data of categorical variables were analyzed by the chi-square test or Fisher’s exact test, when appropriate. Continuous variables were analyzed by the Student’s *t*-test. A *P* value less than 0.05 was considered to be statistically significant. Distributions of variables were reported as percentages and mean ± standard deviation (SD). Statistical analyses were performed using SPSS software (version 15.0; SPSS Inc., Chicago, IL, USA).

## Results

During the 6-year study period, about 165,000 children presented to our pediatric ED. This study included 37 patients (mean age = 10.2 ± 5.5 years), 26 males and 11 females, who presented to the pediatric ED with rhabdomyolysis and met the inclusion criteria. Of the 37 patients, 11 (29.7%) were younger than 7 years. Two of the most commonly presented symptoms among the 37 patients were muscle pain and muscle weakness (83.8% and 73%, respectively). Among them, 25 (67.6%) presented with fever and dark urine was reported in only 5.4% of the patients (Table 1). Three (8.1%) of these 37 patients were admitted to intensive care unit (ICU). The duration of hospital stay was longer in patients with ARF than those without ARF (8.7 ± 6.4 days and 3.4 ± 3.7 days, respectively, *P* = 0.04). All the 37 patients with rhabdomyolysis had a clearly identified single diagnosis based on the patient’s discharge diagnosis or diagnosis through subspecialty follow-up assessments (Table 2). The most common causes of rhabdomyolysis in this sample were infections (n = 22, 59.5%) and the virus was identified in 5 patients (Influenza type B in 4; Coxsackie A10 in 1). For the traumatic causes, traffic accidents (n = 6, 100%) were the major cause and none of these 6 patients were complicated with ARF, including a 4-year-old girl with major trauma. For the 6 patients where involvement was caused by exercise, all of them were in the adolescent group. All cases received a large amount of fluid administration within the first 24 hours, but only one required bicarbonate therapy.

**Table 1:** Demographics and clinical presentations of the children with rhabdomyolysis

		**Non-ARF**		**ARF**		**Total**		
		**(n=34)**		**(n=3)**		**(n=37)**		
**Variables**		**N**	**%**	**N**	**%**	**N**	**%**	**P-value**
Gender	Female	11	32.4	0	0	11	29.7	0.540
	Male	23	67.6	3	100	26	70.3	
Muscular pain	Yes	29	85.3	2	66.7	31	83.8	0.421
Muscular weakness	Yes	26	76.5	1	33.3	27	73	0.172
Muscular swelling	Yes	3	8.8	0	0	3	8.1	1.000
Dark urine	Yes	2	5.9	0	0	2	5.4	1.000
Fever	Yes	22	64.7	3	100	25	67.6	0.537
Admission unit	OU	14	41.2	0	0	14	37.8	0.169
	Ward	18	52.9	2	66.7	20	54.1	
	ICU	2	5.9	1	33.3	3	8.1	

*ARF* acute renal failure, *OU* observation unit, *ICU* intensive care unit.

**Table 2:** Etiologies of patients with rhabdomyolysis and ARF

	**Non-ARF**		**ARF**		**Total**		
	**(n=34)**		**(n=3)**		**(n=37)**		
**Cause**	**N**	**(%)**	**N**	**(%)**	**N**	**(%)**	**P-value**
Trauma	6	(17.6)	0	(0.0)	6	(16.2)	0.035*
Exercise	6	(17.6)	0	(0.0)	6	(16.2)	
Infection	21	(61.8)	1	(33.3)	22	(59.5)	
Metabolic and electrolyte	1	(2.9)	1	(33.3)	2	(5.4)	
Body-temperature change	0	(0.0)	1	(33.3)	1	(2.7)	

*Statistically significant by the chi-square test or Fisher’s exact test when appropriate.

*ARF* acute renal failure.

**Figure 1:**
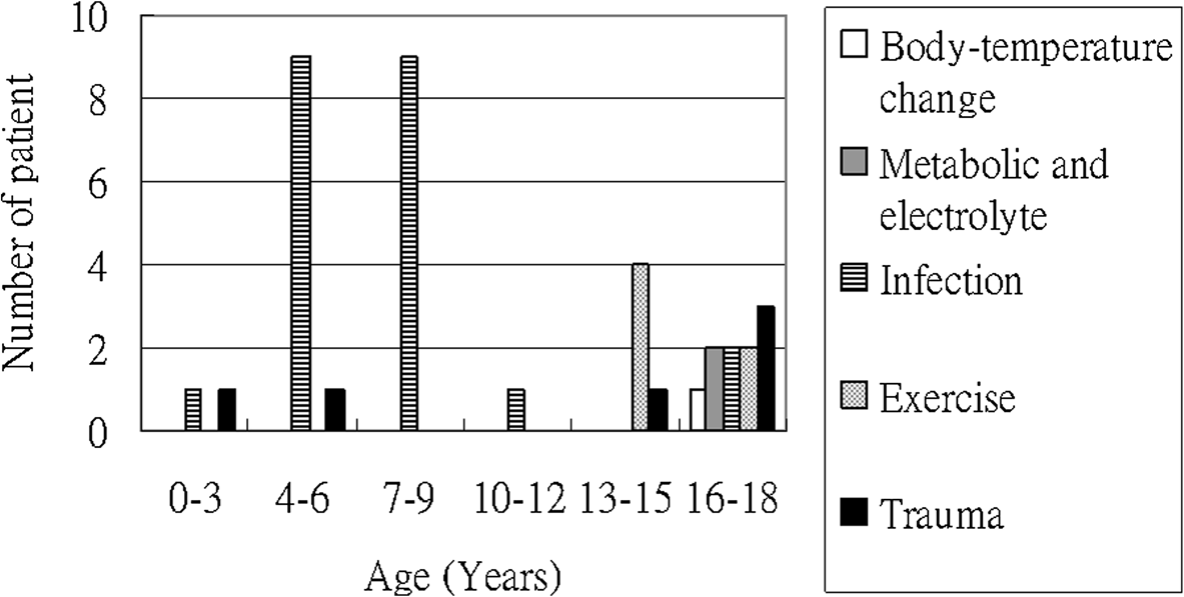
Etiologies of children with rhabdomyolysis in different age groups.

The leading cause of rhabdomyolysis in the 0 to 9-year age group was presumed infection, and the leading cause in the 10- to 18-year age group was trauma and exercise (Figure 1). Moreover, three patients complicated with ARF and no patient needed RRT (Table 3). All 3 patients survived and had normal creatinine levels documented during follow-up visits.

**Table 3:** Details of the pediatric patients with acute renal failure

**Case**	**Presenting symptoms**	**Follow-up findings**	**Cause of rhabdomyolysis**	**Initial CK level (U/L)**	**Peak CK level (U/L)**	**Peak creatinine level (mg/dl)**	**Treatment**	**Outcome**
1	Fever, watery diarrhea, lower limbs muscle pain, weakness	Septic shock	Infection	1224	1224	3.4	Hydration, sodium bicarbonate	No dialysis, alive
2	Fever, weakness, seizure attack (GTC type)	EEG: normal, brain CT: minimal acute subdural hematoma	Heat stroke	1192	6810	2.2	Hydration	No dialysis, alive
3	Abdominal pain, vomiting, lower limbs weakness with spasm	Congenital adrenal gland hyperplasia	Metabolic disease	1429	1429	3.8	Hydration, furosemide	No dialysis, alive

*CK* serum creatine kinase, *GTC* generalized tonic-clonic seizure, *EEG* electroencephalography, *CT* computed tomography.

The peak serum CK level in those 37 patients was 9825.1 ± 23079.1 U/L, there was no difference between the ARF and non-ARF groups (Table 4). The serum level of myoglobin was checked in half of our study patients and the peak value in those 16 patients was 1634.8 ± 2334.4 ng/mL. There was no significant difference between the in ARF and non-ARF groups. BUN and Creatinine were significantly higher in the ARF group compared to the non-ARF group.

**Table 4:** Comparison of laboratory tests of patients between AFR and non-ARF groups

	**Non-ARF**			**ARF**			***P-***
							**value**
	**N**	**Mean**	**SD**	**N**	**Mean**	**SD**	
WBC (× 10^9^/L)	33	7796.1	5734.0	3	17663.3	9229.2	0.056
BUN (mg/dl)	19	8.6	2.9	3	36.6	14.9	0.001*
Creatinine (mg/dl)	31	0.6	0.3	3	3.1	0.8	<0.001*
ALT (U/L)	19	121.8	254.0	3	71	24.6	0.218
AST (U/L)	11	285.1	354.6	3	80.0	40.1	0.555
Sodium (mmol/L)	28	138.8	2.1	3	135.7	7.4	0.392
Potassium (mmol/L)	29	3.8	0.5	3	4.0	0.5	0.666
CK-peak (U/L)	34	10413.6	24001.2	3	3154.3	3167.6	0.814
CK-initial (U/L)	16	2809.2	3436.4	3	1281.7	128.6.5	0.712
Myoglobin-peak (ng/mL)	15	1675.1	2410.5	1	1030		0.75
Myoglobin-initial (ng/mL)	9	1262.2	1585.2	1	1030		0.60

*Statistically significant by Student’s t-test.

*WBC* white blood count, *BUN* blood urea nitrogen, *ALT* alanine aminotransferase, *AST* aspartate aminotransferase, *CK* creatine phosphokinase.

## Discussion

Rhabdomyolysis is a potentially life-threatening syndrome that can develop from a variety of causes; the classic triad of symptoms includes muscle pain, weakness, and dark urine and may not always be present in children [4-10,13]. In 2006, an adult study showed that 45% presented with myalgia, 38% had muscle weakness and only 3.6% noted dark urine [12]. In our study, we recorded 83.8% presented with myalgia, 73% had muscle weakness but dark urine was observed in only 5.4%. There was only one patient presented with the classic triad of symptoms. In our study, we also found the male to female ratio of pediatric rhabdomyolysis was 2.3:1. The result was similar to the two other studies of rhabdomyolysis which the male to female ratio were 2:1 and 4:1, respectively. [11,12].

The etiologies of rhabdomyolysis can be classified as hereditary and acquired. The acquired causes are further classified as traumatic and non-traumatic [14]. In pediatric patients, the most common causes are viral myositis, trauma, connective tissue disorders, seizure, physical exertion, and drug overdose [11,12]. In our series, infections accounted for more than half (59.5%) of the causes of rhabdomyolysis, followed by trauma (16.2%) and exercise (16.2%). In patients that were 9 years old or younger, infections was the most common cause of rhabdomyolysis and accounted for 19 of 21 patients in this age group. However, the most common cause of rhabdomyolysis in patients older than 9 years was physical exertion. Rhabdomyolysis has been reported to be associated with a variety of viral infections, including influenza, [15,16] Coxsackie virus, human immunodeficiency virus (HIV), echovirus and cytomegalovirus [17]. In our series, the definite viral infection was identified in 5 patients (influenza type B in 4, Coxsackie A10 in 1) and none of them were complicated with ARF. In these 6 patients that presented with rhabdomyolysis caused by trauma, traffic accident was the major cause and none of these 6 patients were complicated with ARF.

ARF is a major life-threatening complication of rhabdomyolysis and requires immediately adequate managements. Previous studies reported the rates of ARF secondary to rhabdomyolysis range from 17-35% in adults and from 42-50% in children [18-21]. However, in the recently larger pediatric studies reported by Mannix et al [12] and Wu et al [11] it was shown that the rate of ARF with pediatric rhabdomyolysis ranged from 5–8.7 %. The indications for RRT include severe and resistant hyperkalemia, an abrupt increase in potassium levels, persistent metabolic acidosis, and ongoing ARF despite conservative treatment [22-24]. Based on previous studies [11,12], the rate of RRT was from 1.6-2.9% in children with rhabdomyolysis. In our study, we found a lower rate (8.1%) of ARF, and our series showed no one treated with RRT. The outcome of patients examined in this study was considered to be good.

To prevent ARF, the early recognition and appropriate treatment of rhabdomyolysis is vital. Furthermore, the predictors of ARF have been investigated in previous studies [3,5,10-12,25]. Serum CK represents a more reliable marker than blood myoglobin for the diagnosis and assessment of the severity of rhabdomyolysis because it lasts for longer periods than blood myoglobin [3]. However, elevated serum CK has not been shown to well correlate with the severity of ARF, [5,25] although some studies showed a predictive correlation between CK and ARF [10,11]. Our study also indicates that the initial and peak value of CK is not a good predictor of ARF. Below are some probable reasons to explain that the peak level of CK and myoglobine is higher in the non-ARF group as compared to the ARF group in our study. First, there was a small sample size included in our study which only contained 3 patients with ARF. Second, the laboratory data were not regularly followed up in all patients, and this may lead to difficulty in determining the definite peak data. Third, the pathophysiology of rhabdomyolysis is heterogeneous and this may cause the different appearance of laboratory data based on different etiologies of rhabdomyolysis. Moreover, the definite relationship between peak CK or myoglobin and ARF caused by different etiologies of rhabdomyolysis is not clear enough. A recent adult study, conducted by Kasaoka et al [25], suggested that the serum myoglobin level on admission did not predict ARF, but that the peak value of serum myoglobin might be a predictive factor of ARF. The best cutoff value for serum myoglobin was 3865 ng/mL. But due to the relatively small sample size (30 patients), they could not make a definitive conclusion. However, our study showed no significant differences in laboratory findings that could help to predict ARF earlier in patients with rhabdomyolysis.

There is still a lack of randomized controlled trials for the best treatment in children with rhabdomyolysis to be conducted till now. However, regardless of underlying etiologies, the important aspects of treatments for rhabdomyolysis are considered to be conservative [6,13,26]. Clinically, managements may include prompt and aggressive fluid resuscitation, prevention of progressing to ARF, early correction of potentially lethal electrolyte disturbance, correction of severe metabolic acidosis, and managements of other coexisting complications [6,13,26]. In addition, for adults, the addition of mannitol and bicarbonate after the initial fluid resuscitation to prevent ARF has been recommended (especially for crush injury) in some studies [13,27,28]. But, the role of mannitol or bicarbonate in the treatment for rhabdomyolysis in children remains controversial [26]. Although causes of rhabdomyolysis are wide variety and the definite pathophysiology and development of ARF are complicated, the main therapeutic intervention to avoid ARF should be fluids and hydration [6,13,26]. Therefore, once the diagnosis of rhabdomyolysis in children is made in the ED, aggressive intravenous fluid rehydration will be indicated.

The present study has a number of limitations. First, in a retrospective single center review of medical records, some details of history and physical examinations may not be rigorously documented. Second, the relatively small sample size may fail to address definitely predictive factors of ARF. Finally, the laboratory data were not regularly followed up in some patients and the definite peak data was therefore difficult to record. These limitations may have led to some bias in analyzing the clinical spectrum of rhabdomyolysis in children who presented to the ED.

## Conclusion

Infection was the major cause of rhabdomyolysis in children younger than 10 years; however, the leading causes in the teenager group were trauma and exercise. In our study, the classic triad of symptoms of rhabdomyolysis included myalgia, weakness and dark urine were rarely presented and the incidence of ARF associated with rhabdomyolysis was 8.1%. Although our study did not identify any unique predictors of ARF or need for RRT, the prognosis of rhabdomyolysis was good with appropriate management.

## Competing interest

There is no competing interest related to this study.

## Authors’ contributions

CYC and WCY reviewed the medical records, analyzed and interpreted the data, and drafted the manuscript; LLZ and KHW interpreted the data, and drafted the manuscript. YJC analyzed and interpreted the data. HPW designed and oversaw the study, interpreted the data, and revised the manuscript. All authors have read and approved the final manuscript for publication.

## Pre-publication history

The pre-publication history for this paper can be accessed here:

http://www.biomedcentral.com/1471-2431/13/134/prepub
